# Perennial Cotton Ratoon Cultivation: A Sustainable Method for Cotton Production and Breeding

**DOI:** 10.3389/fpls.2022.882610

**Published:** 2022-06-06

**Authors:** Xin Zhang, Qian Yang, Ruiyang Zhou, Jie Zheng, Yan Feng, Baohong Zhang, Yinhua Jia, Xiongming Du, Aziz Khan, Zhiyong Zhang

**Affiliations:** ^1^Henan Collaborative Innovation Centre of Modern Biological Breeding, Henan Institute of Science and Technology, Xinxiang, China; ^2^Foreign Languages College, Henan Institute of Science and Technology, Xinxiang, China; ^3^College of Agriculture, Guangxi University, Nanning, China; ^4^State Key Laboratory of Cotton Biology, Institute of Cotton Research, Chinese Academy of Agricultural Science, Anyang, China; ^5^Hainan Yazhou Bay Seed Laboratory, Sanya, China; ^6^National Nanfan Research Institute (Sanya), Chinese Academy of Agriculture Sciences, Sanya, China; ^7^Department of Biology, East Carolina University, Greenville, NC, United States

**Keywords:** *Gossypium* (cotton), heterosis, indeterminate, male-sterile, stub

## Abstract

Cotton production is challenged by high costs with multiple management and material inputs including seed, pesticide, and fertilizer application. The production costs can be decreased and profits can be increased by developing efficient crop management strategies, including perennial cotton ratoon cultivation. This review focuses on the role of ratoon cultivation in cotton productivity and breeding. In areas that are frost-free throughout the year, when the soil temperature is suitable for cotton growth in spring, the buds of survived plants begin to sprout, and so their flowering and fruiting periods are approximately 4–6 weeks earlier than those of sown cotton. Due to the absence of frost damage, the ratoon cotton continues to grow, and the renewed plants can offer a higher yield than cotton sown in the following season. Moreover, ratoon cultivation from the last crop without sowing can help conserve seeds, reduce labor inputs, and reduce soil and water loss. In this review, the preservation of perennial cotton germplasm resources, the classification and genome assignment of perennial species in the cotton gene pools, and effective strategies for the collection, preservation, identification, and utilization of perennial cotton germplasms are discussed. Ratoon cultivation is the main driver of cotton production and breeding, especially to maintain male sterility for the utilization and fixation of heterosis. Ratoon cultivation of cotton is worth adopting because it has succeeded in Brazil, China, and India. Therefore, taking advantages of the warm environment to exploit the indeterminant growth habit of perennial cotton for breeding would be an efficiency-increasing, cost-saving, and eco-friendly approach in frost-free regions. In the future, more attention should be given to ratooning perennial cotton for breeding male-sterile lines.

## Introduction

Cotton is an industrial textile crop and the most widely grown natural fiber crop (Zhang et al., 2022). Excellent varieties are the basis for high cotton yields, especially those developed in the 1990's, when the breeding of Bt cotton saved cotton production, which was almost destroyed by bollworm. This advancement also reduced the use of chemical pesticides, saving both human and material resources and contributing to environmental protection and the ecological balance. It was therefore rapidly promoted and applied in production. However, Bt-transgenic insect-resistant cotton only has a particular effect on some *Lepidoptera* pests and is not very effective against aphids, sooty mites, and other pests that are currently causing severe damage in production (Bergman, 1985; Silva et al., 2008; Wan et al., 2017). In addition, as labor inputs are higher for cotton than food crops, while the economic efficiency is low, and cotton is planted in drought-affected and saline areas as well as mudflats, the breeding of cotton varieties with multistress resistance has important and far-reaching significance.

Given the narrow genetic basis of high-yield cotton varieties worldwide and their high degree of homogeneity, as well as the controversy over the ecological safety risks associated with genetically modified cotton, it is extremely important to use semiwild lines of cultivated species and wild species, which make up the majority of *Gossypium*, as germplasm resources to expand the genetic basis of annual cotton varieties (Teravanesyan and Belova, 1970; Wang, 2007; Migicovsky and Myles, 2017). In the future, it will be necessary to utilize the excellent traits of perennial species and their underlying genes.

The high regeneration potential of cotton has long been recognized, with examples of ratoon cotton being grown in Georgia as early as 1786 (Seabrook, 1844). In 1961, Stroman proposed ratooning F_1_ plants in Peru to harvest more than one crop of F_2_ seeds (Stroman, 1961). In 1968, Weaver proposed that ratooning male-sterile plants would provide an excellent way to produce F_1_ seeds (Weaver, 1968). In the practice of cotton breeding, ratoon cotton could be used to extend the time in which germplasms can be utilized for more than one season (Muhammad et al., 2015). In tropical cotton production areas, the most economical way to take advantage of ratoon cotton is to produce hybrid cotton seeds with high quality and low cost by ratooning male-sterile lines because if ratoon cotton is used for lint production in the tropics, its economic benefit is far lower than that of hybrid seed production and even worse than that of cotton sown annually in temperate areas.

## Perennial Conservation of *Gossypium* Species and Their Classification

**Figure 1:**
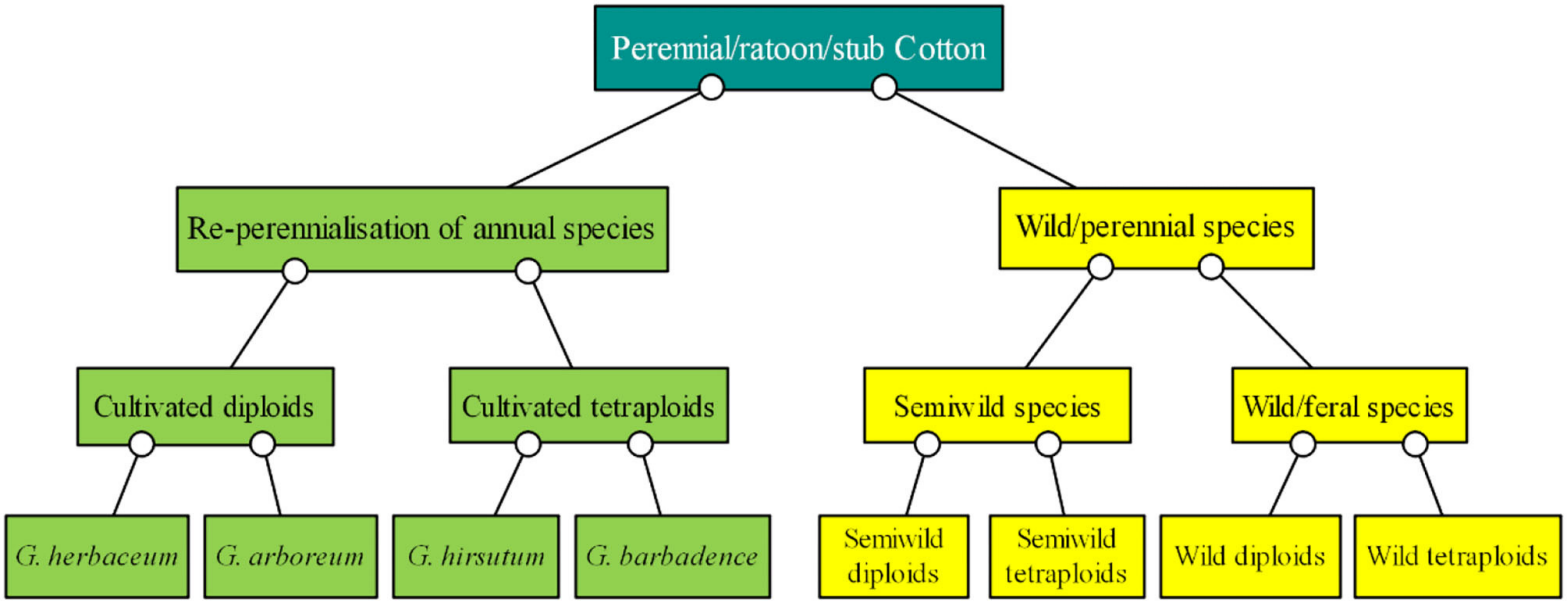
Classification of ratoon cotton based on cultivars, semiwild species, wild/feral species, and chromosome ploidy. The green boxes show the cultivated species, and the yellow boxes show the wild species.

Of the *Gossypium* species, four are cultivated species and the others are perennial wild species (Figure 1). The basis of cotton breeding is the collection and preservation of genetic germplasms, which provide an essential foundation to improve and sustain cotton production (Zhang et al., 2012a). Specifically, the following extraordinary accessions are required for perennial preservation: (1) wild cotton species with short-day flowering behavior, from which it is hard to obtain seeds (Percy et al., 2014); (2) nullisomic, monosomic, telomeric, trisomic, and translocation lines and other cytogenetic stocks of cotton (Kiranga, 2013), the fruiting rate of which is low and the identification of which is difficult and time-consuming; (3) cotton plants infected by some kinds of pathogens, the status of which should be maintained for a long time for research (Mihail et al., 1987; Seo et al., 2006); and (4) cotton hybrids or backcross generations such as F_1_, F_2_, and BC_1_F_1_ generations, which can be commonly used for only a year but the generations of which can be repeatedly used over many years through perennial growth (D'Eeckenbrugge and Lacape, 2014).

### Global Overview of Perennial Germplasms and Perennial Conservation of Cotton

Worldwide, *Gossypium* germplasms with different ecological niches have much morphological, agronomic, physiological, and genetic variability that is conserved *in situ* at centers of cotton origin (Castro et al., 2016) and preserved *ex situ* with a large number of accessions in eight major countries with extensive cotton germplasm collections, namely Australia, Brazil, China, France, India, Russia, the USA, and Uzbekistan (Abdurakhmonov, 2007; Rahmat et al., 2014; Boopathi and Hoffmann, 2016), attaching great importance to the preservation of perennial germplasms. It is worth noting that more than 20,000 cotton germplasm accessions are preserved in Uzbekistan, making that the most extensive collection in the world. Although cotton is now rarely grown in France, it is commendable that more than 3,000 accessions, including approximately 1,000 wild accessions, are preserved in CIRAD (Coopération Internationale en Recherche Agronomique pour le Développement), a French publicly supported agency that specializes in tropical and Mediterranean agriculture (Campbell et al., 2010).

At present, China, India, and the USA are the three largest cotton-producing countries. Of the ~10,000 accessions preserved in the National Cotton Germplasm Collection (NCGC) of the USA, 581 are wild germplasms. Most of the accessions, including photoperiodic germplasms and perennial accessions, are perennially grown at the tropical Cotton Winter Nursery (CWN) in Tecoman, Colima, Mexico (Wallace et al., 2009; Percy et al., 2014). In India, the bank of the Central Institute for Cotton Research has collected a total of 10,227 accessions, including 26 wild species and 32 perennial forms (Boopathi et al., 2014). Although China is not an origin center of cotton, most of the 8,868 accessions, including 32 wild forms, were collected from China, and 2,236 accessions were introduced from 52 foreign countries. The Sanya National Research Station of Wild Cotton Germplasm, which is located within the tropics of China, has 391 wild accessions that are perennially grown for conservation (Jia et al., 2014). In addition, Mexico, Pakistan, and other cotton-planting countries have also collected many germplasms.

### Gene Pools and Genome Assignment of Perennial *Gossypium* Species

**Figure 2:**
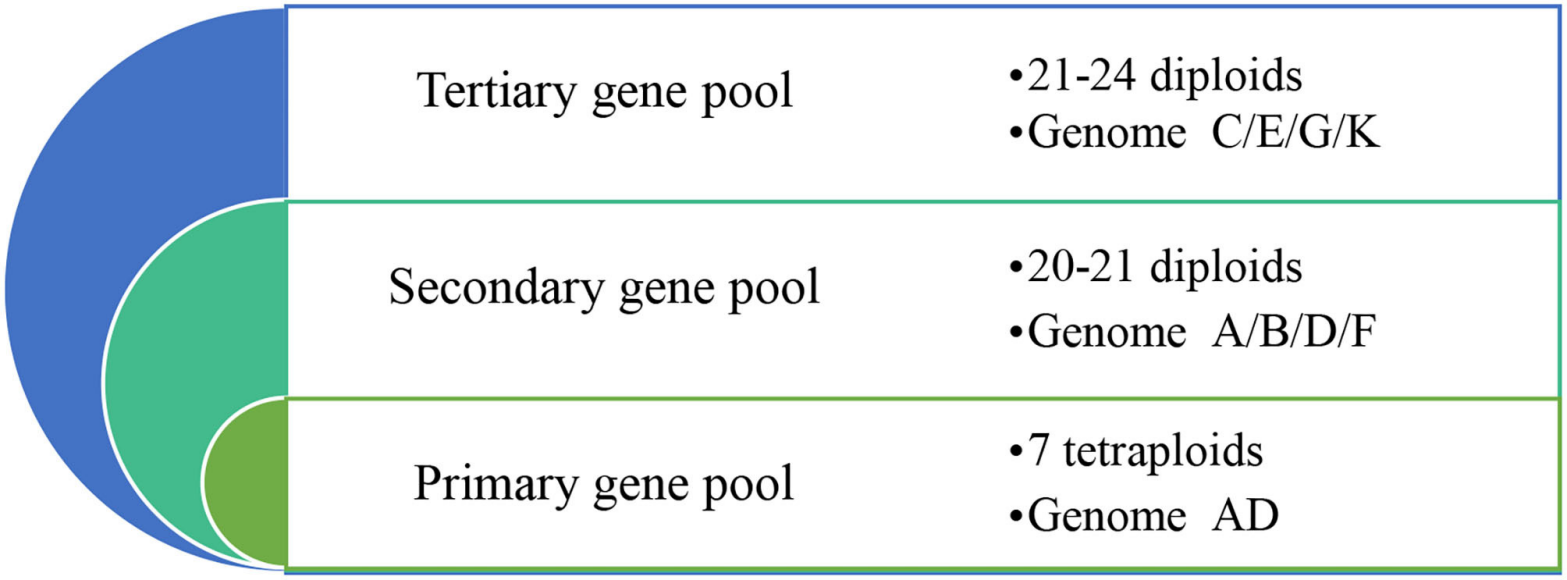
The primary, secondary, and tertiary gene pools based on their genetic relationship with upland cotton. The primary, secondary, and tertiary cotton (*Gossypium*) gene pools are shown from the inside circle to the outer ring. The farther away the primary gene pool is the further the genetic approachability is from the tetraploids, and the richer the genetic diversity.

At least 48 species of *Gossypium*, including 7 tetraploid (2*n* = 4*x* = 52) species, 41-45 diploid (2*n* = 2*x* = 26) species, and other wild species, originate from arid to semiarid regions within the tropics and subtropics (Wendel and Grover, 2015; Shim et al., 2018; Wang et al., 2018). According to the genetic relationship with upland cotton, all the cotton species can be classified into primary, secondary, and tertiary gene pools. Of the 7 tetraploid species (genome AD), *G. hirsutum* and *G. barbadense* are mainly cultivated worldwide, so all tetraploid species are in the primary gene pool. Based on the relative genetic approachability and utility of species to improve *G. hirsutum* and *G. barbadense*, 20–21 diploid species (genome A/D/F/B) and 21–24 diploid species (genome C/E/G/K) have been classified into secondary and tertiary gene pools, respectively (Figure 2).

*Gossypium* species with genomic assignments and geographical origins in the primary, secondary, and tertiary cotton gene pools are detailed in Table 1. Responding to the diverse geographic and ecological conditions of frost-free regions, wild cotton species show a broad adaptation range from herbaceous perennial diploid species with a fire-/dry-adapted biseasonal growth pattern in northwest Australia to small cotton trees dropping their leaves to avoid the effects of the dry season in southwest Mexico (Campbell et al., 2010). Therefore, it is widely believed that the extensive genetic diversity within wild cotton increases their opportunities for evolutionary adaptation that reduces their genetic vulnerability to the changing harmful environments (Boopathi and Hoffmann, 2016).

**Table 1:** *Gossypium* species with genome assignment and geographic origin in the primary, secondary, and tertiary gene pools.

**Gene pool**	**Genome**	**Presently recognized species in *Gossypium* [Genome assignment, geographic origin]**
Primary (7 tetraploids)	AD (7 species)	*G. hirsutum* [(AD)_1_, Central America], *G. barbadense* [(AD)_2_, South America], *G. tomentosum* [(AD)_3_, Hawaiian Islands], *G. mustelinum* [(AD)_4_, Brazil], *G. darwinii* [(AD)_5_, Galapagos Islands], *G. ekmanianum* [(AD)_6_, Dominican Republic], *G. stephensii* [(AD)_7_, Wake Atoll]
Secondary (20–21 diploids)	A (2 species)	*G. herbaceum* [A_1_, Southern Africa] (*subs. africanum* [A_1−a_, Southern Africa]), *G. arboretum* (syn. *G. aboreum*) [A_2_, Indus valley, Madagascar]
	D (13–14 species)	*G. thurberi* [D_1_, Mexico], *G. armourianum* [D_2−1_, Mexico], *G. harknessii* [D_2−2_, Mexico], *G. davidsonii* [D_3−d_, Mexico], *G. klotzschianum* [D_3−k_, Galapagos Islands], *G. aridum* [D_4_, Mexico], *G. raimondii* [D_5_, Peru], *G. gossypioides* [D_6_, Mexico], *G. lobatum* [D_7_, Mexico], *G. trilobum* [D_8_, Mexico], *G. laxum* [D_9_, Mexico], *G. turneri* [D_10_, Mexico], *G. schwendimanii* [D_11_, Mexico], {*G*. sp. nov. [D_12_, Mexico]}^a^
	F (1 specie)	*G. longicalyx* [F_1_, Africa]
	B (4 species)	*G. anomalum* [B_1_, Africa (Angola, Namibia)], *G. triphyllum* [B_2_, Namibia in Africa], *G. capitis-viridis* [B_3_, Cape Verde Islands], *G. trifurcatum* [B, Somalia]
Tertiary (21–24 diploids)	E (4–7 species)	*G. stocksii* [E_1_, East Africa, Arabia], *G. somalense* [E_2_, NE Africa], *G. areysianum* [E_3_, Arabia], *G. incanum* [E_4_, Arabia], {*G. benadirense* [E, Somalia, Ethiopia, Kenya]}^b^, {*G. bricchettii* [E, Somalia]}^c^, {*G. vollesenii* [E, Somalia]}^d^
	C (2 species)	*G. sturtianum* [C_1_, Australia] (var. *nandewarense* [C_1N_, Australia]), *G. robinsonii* [C_2_, Australia]
	G (3 species)	*G. bickii* [G_1_, Australia], *G. australe* [G_2_, Australia], *G. nelsonii* [G_3_, Australia]
	K (12 species)	*G. exiguum* [K_1_, Australia], *G. rotundifolium* [K_2_, Australia], *G. populifolium* [K_3_, WA Australia], *G. pilosum* [K_4_, WA Australia], *G. marchantii* [K_5_, Australia], *G. londonderriense* [K_6_, Australia], *G. enthyle* [K_7_, Australia], *G. costulatum* [K_8_, Australia], *G. cunninghamii* [K_9_, Northern NT Australia], *G. pulchellum* [K_10_, WA Australia], *G. nobile* [K_11_, Australia], *G. anapoides* [K_12_, Australia]

*The data came from Wendel and Grover (2015), Shim et al. (2018), and Wang et al. (2018). The farther the genome from the primary gene pool is, the further the genetic approachability. ^a^ No formal name yet, ^b,c,d^no living materials of these species have been available for further study (Wang et al., 2018)*.

### Efficient Strategies of Collecting, Conserving, and Characterizing Perennial Cotton Germplasm

Perennial cotton germplasms can be widely collected through exploration in tropical origin centers or via exchange with other gene banks. Perennial cotton can be conserved *in situ* or *in vivo* in tropical fields. After harvesting enough seeds, the seeds can be preserved in the gene bank. Moreover, perennial cotton germplasm can be preserved as perennial roots and cuttings via grafting and tissue culture in greenhouses or laboratories. Most traits can be evaluated in tropical areas, and some abiotic or biotic stress responses can be characterized in greenhouses. Molecular biological methods, such as genomics, phenomics, and molecular markers, can be used to improve the efficiency of characterizing perennial cotton germplasms.

## Ratoon Cultivation of Perennial and Annual Cotton for Breeding

**Figure 3:**
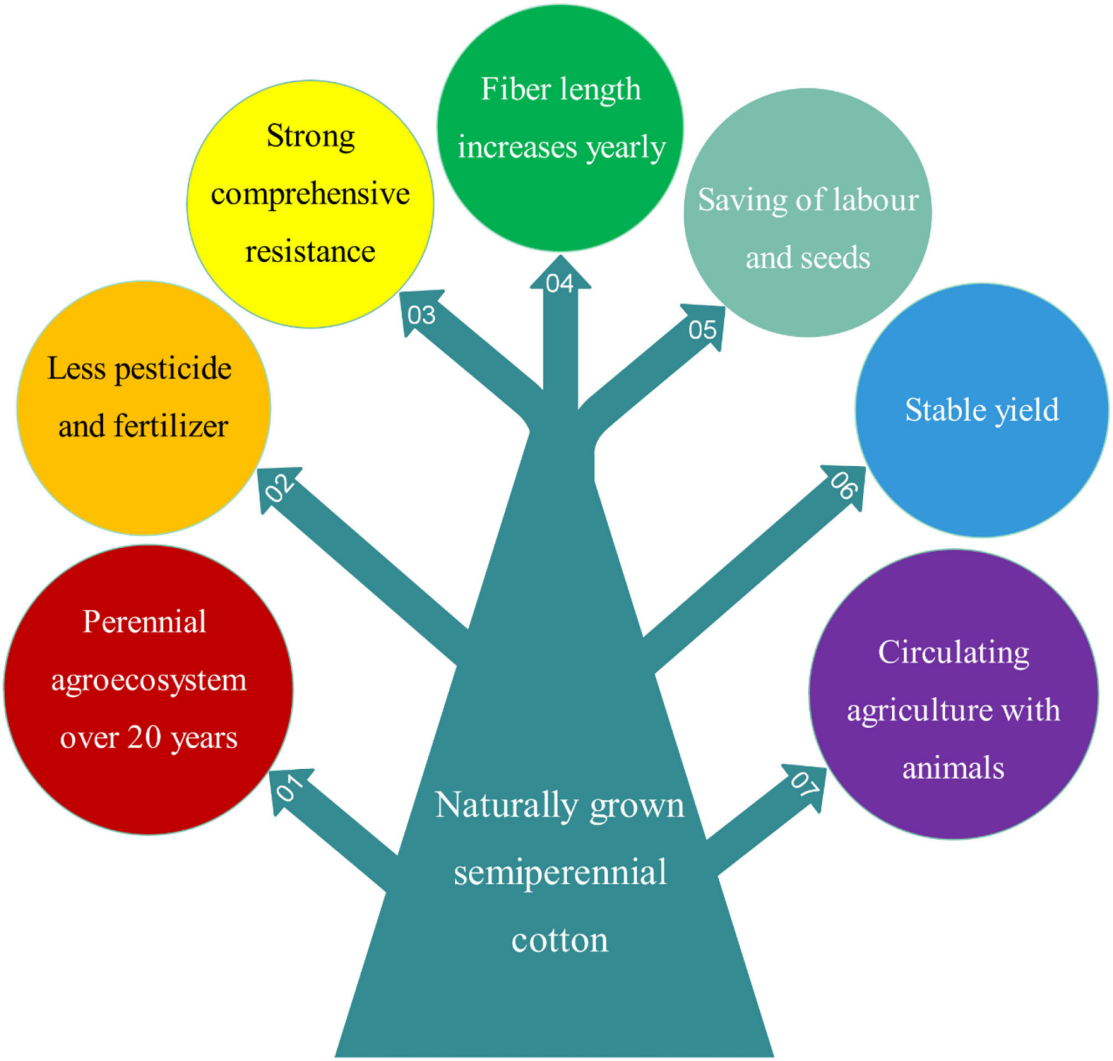
An overview of the seven main advantages of naturally grown semiperennial (semiwild) cotton lines compared to annual cultivars.

Both perennial and annual cotton species can be cultivated perennially in greenhouses or frost-free regions, and they should be cultivated by ratooning (Figure 1). Otherwise, yields of unratooned perennial cotton forms are meager. Many studies show that the service life, pesticide and fertilizer usage, stress resistance, and yield stability of semiwild races are better than those of annual species in the ratoon cultivation of cotton (Figure 3), and the annual species could be ratoon cultivated for ~3 years (Evenson, 1970; Plucknett et al., 1970; Chen et al., 2010a,b; Zhang et al., 2020a,b). It is worth noting that ratoon cultivation of hybrids between upland cotton and sea island cotton has been performed, which was helpful for fixing the interspecific heterosis of cotton (Komala et al., 2018a,b,c). The benefits of ratoon cultivation of perennial cotton mainly come from three features: (1) the perennial root system, which can save costs for seed and tillage, shorten the vegetative growth period, and provide monetization from an earlier harvest; (2) the low pruned stem, which can reduce plant height and increase the number of fruiting branches; and (3) infinite inflorescences, which can extend the flowering period and increase boll number and yield (Table 2).

**Table 2:** Experimental evidence for the benefits of ratoon cultivation of perennial cotton.

**Main feature**	**Benefits**	**Reference**
Perennial root system	Conserved costs for seed and tillage	Macharia (2013); Zhang et al. (2013)
	Shortened the vegetative growth period	Chamy (1979); Komala et al. (2019)
	Monetizing from an earlier harvest	Mubvekeri et al. (2014)
Low pruned stem	Reducing plant height	Reddy and Thimmegowda (1997a); Macharia (2013)
	Increasing the number of fruit branches	Reddy and Thimmegowda (1997b); Chen (2008)
Indefinite inflorescences	Extended the flowering period	Plucknett et al. (1970); Macharia (2013)
	Increasing boll number and yield	Chen et al. (2008); Zhang et al. (2015a)

### Perennial Cropping Methods for Ratoon Cotton

The ratoon cropping system achieves increased cotton yield with less labor input and decreases the cost of producing F_1_ generation cotton seeds (Bergman et al., 1983). Zhang et al. summarized three methods of ratoon cotton cropping systems, including ratooning semiwild cotton used for perennial cropping, ratooning annual cotton cultivars for perennial cropping, and ratooning annual cotton cultivars for biannual cropping (Zhang et al., 2020a). However, there is abundant evidence that failure to control pests effectively can lead to a severe impact on the yield of ratoon cotton (Flint et al., 1980). Although the perennial cultivation of ratoon cotton has declined and is even banned in some countries (Templeton, 1925; Evenson, 1970; Plucknett et al., 1970; Morris, 1973), ratooning for the second fruiting cycle with an increased yield in the same season can be used in areas with closed season legislation and has great potential and prospects in cotton production (Table 3).

**Table 3:** Comparisons among the three cropping systems of ratoon cotton.

**Comparison items**	**Common cropping**	**Perennial cropping**	**Biannial cropping**
Service life	One season	Usually 3 years	Two seasons
Annual yield	Low	Medium	High
Risk of pests and diseases	Low	High	Medium
Adaptability to irregular rainfall	Medium	High	Low
Annual labor costs	Medium	Low	High
Annual costs of tillage and seeds	Medium	Low	High
Annual fertilizer demand	Medium	Low	High
Weed pressure	Medium	Low	High
Loss of soil, nutrients and water	Medium	Low	High

*The data obtained from Zhang et al. (2020a)*.

### Key Measures to Obtain a High Yield of Ratoon Cotton

Only the annual branches (new branches formed within 1 year) can produce fruits, so pruning, fertilization for rejuvenation, and other proper techniques to increase sprout formation are particularly important for the second fruiting cycle of ratoon cotton in the tropics (Gutstein, 1969; Reddy and Thimmegowda, 1997a; Azevedo et al., 2000; Chen et al., 2010c; Khader and Prakash, 2014; Vukicevich et al., 2016); pruning time and pruning height are the most critical factors for proper fruiting (Reddy and Thimmegowda, 1997b). Moreover, some practical techniques have been proposed for ratoon cultivation of cotton, including winter management, pest control, fertilization, and hormone regulation, based on experimental results or experience (Sachs and Zilkah, 1985).

**Figure 4:**
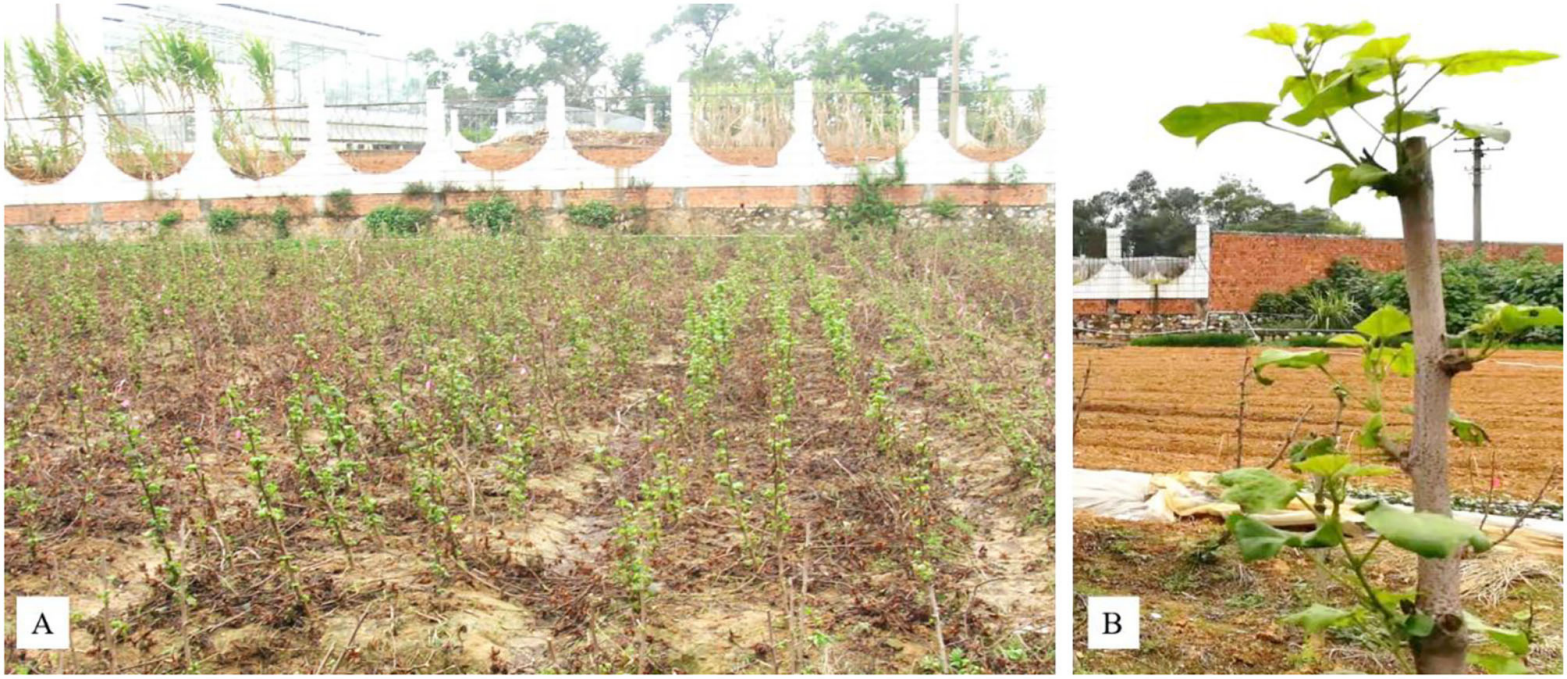
Ratoon cotton planted in the experimental field on the campus of Guangxi University, Nanning, China. Photos of the population **(A)** and a single plant **(B)** after the main stem is pruned.

The main stems of cotton crops were cut at different heights above ground level after harvest, and the remaining stumps regenerated new shoots at the beginning of the next rainy season to provide ratooned cotton crops (Figure 4). Macharia studied the effect of cutting-regenerated cotton sown after harvest at heights of 5, 10, and 15 cm above the ground and showed that the average kapas yields of the three cultivars were 344.0, 381.3, and 408.7 kg/ha, respectively (Macharia, 2013), which showed that the cutting height can be further improved. In addition, the effect of the cutting height on various cultivars was different. When the cutting height of the cultivars “HART 89M” and “F962” was 15 cm, the seed cotton yields were the highest, while “A540” had the highest seed cotton yield when the cutting height was 10 cm. However, because the yield of ratooned crops increased with the proper cutting height, the optimum cutting height may be higher than 15 cm. Moreover, some studies showed that a high yield of ratoon cotton could not be sustained after the third year due to deep pruning at 5–15 cm above the soil level. In particular, it must be noted that the wound left on ratoon cotton by pruning easily becomes a point of water loss and invasion by pests and diseases. Lumps occur at the plant base and necrosis occurs at the top of the stub under the wound, which is the result of no-wax sealing or dressing of the damage.

### Grafting Annual Cotton to Achieve Perennial Cultivation in Subtropical Frost-Free Areas

In subtropical frost-free areas, annual cotton cultivars with high yield and good fiber quality grafted onto perennial species with strong resistance to stress, such as drought and low temperature, are conducive to perennial cultivation (Zhang et al., 2013, 2022). Annual cotton cultivated into perennial forms by this method could undergo more growth cycles than normal ratooning crops due to the use of perennial species as rootstocks (Zhang et al., 2022). In addition, the utilization of grafted and perennially maintained male sterility for the production of cotton hybrids has the advantage of eliminating the need for maintainer lines in standard methods and saving agricultural material for sowing, thus reducing the cost of seed production for cotton hybrids. The use of wild cotton as a rootstock can expand the geographical range of seed production using ratooned cotton (Zhou, 2016). Experimental proof for grafting and its role in increasing yield with minimum effort is summarized in Table 4.

**Table 4:** Experimental proof for grafting and its role in increasing yield with minimum effort.

**Roles of grafting**	**Specific role of grafting**	**Reference**
Improving resistance to biotic stress	Improving the resistance of scion to *Verticillium* wilt	Lou (2010); Zhang et al. (2012b)
	Improving the resistance of scion to leaf curl disease	Ullah et al. (2014); Nawaz et al. (2019)
	Improving the resistance of scion to bollworm	Rui et al. (2005); Jin (2013)
Improving tolerance to abiotic stress	Improving the overwintering ability of scion	Zhang et al. (2013)
	Improving salt tolerance of scion	Kong et al. (2012); Qiu et al. (2015)
	Improving drought tolerance of scion	Luo et al. (2019)
Reducing the cost of producing F_1_-seeds	Maintaining sterility for heterosis utilization	Zhang and Zhou (2009); Zhang et al. (2015b)
	Omitting corresponding maintainer	Zhang et al. (2013); Zhou (2016)
	Increasing the yield of scion under *Verticillium wilt* stress	Hao et al. (2010); Zhang et al. (2018)

### Producing Commercial F_1_ Hybrid Cotton Seeds by Ratoon Cultivation

Producing inexpensive hybrid F_1_ cotton seeds with high purity and heterosis has significance in commercial breeding. This production approach offers the highest economic potential for cultivating ratoon cotton in frost-free regions (Zhang et al., 2020a, 2022). There was no difference in yield and fiber quality between the hybrid F_1_ of the male-sterile line “Dong A” with and without ratoon cultivation crossed with the same male parent (Zhang and Zhou, 2009; Zhang et al., 2010). Therefore, breeding out cotton male-sterile lines with strong overwintering survival ability and fine comprehensive traits, and taking advantage of ratoon cultivation to maintain its sterility for producing hybrid seeds could reduce the current production cost of hybrid seeds (Zhang et al., 2015a; Zhou, 2016). This method does not require plowing the land and sowing the seeds each year, reducing the cost of raw materials for production and labor.

Furthermore, rouging and sister crossing would be avoided, which could simplify the breeding procedures of producing hybrid F_1_ cotton seeds and reduce the cost of breeding GMS lines, and the seed yield and purity of the hybrid cotton seeds could be improved. However, in the case of large-scale production of hybrid F_1_ cotton seeds, it is worth noting that artificially bred bees can be used, which can further enhance the purity (Zhang et al., 2015a). Additionally, high temperatures may cause some problems in producing hybrid F_1_ cotton seeds, such as trace fertile pollen occurring on male-sterile cotton in the tropics, which could be addressed by increasing heat tolerance through breeding.

### Hybrids That Can Support Ratoon Cropping Patterns in Cotton

Hybrids can be used in ratoon cotton cropping for three objectives: (1) fixing heterosis for lint production, which requires assessment of the ratoon fiber yield and quality; (2) breeding new varieties for ratoon cropping, which requires the assessment of the combined ability of hybrids and their heterotic effects and (3) producing F_2_ seeds, which requires the analysis of the ratooning ability, genetic variability, and heritability of seed yield in the F_2_ generation (Table 5).

**Table 5:** Hybrids used in ratoon cotton cropping.

**Main objectives**	**Support approach**	**Reference**
Lint production	Fixing heterosis	Reddy and Thimmegowda (1997b); Komala et al. (2018b,d)
	Assessing ratooning ability of fiber trait	Komala et al. (2018d,e)
Breeding new varieties	Assessing combination ability	Komala et al. (2018c,f, 2019)
	Assessing heterotic effects	Komala et al. (2018c,d)
Producing F_2_-seeds	Assessing ratooning ability of seed yield	Komala et al. (2018c,d)
	Analysising genetic variability and heritability	Komala et al. (2018g)

## Ratooning Perennial Cotton for Genetic Research and Breeding

Genetic studies supporting ratoon cropping mainly include the following: (1) investigating heritability such as immortalizing segregated genetic populations and assessing the genetic stability of agronomic traits, (2) studying the variability of traits across generations, such as observing hybridization variation and variability by γ-mutagenesis over many years, and (3) researching the diversity of germplasm resources, such as revealing the origin, distribution and evolution, resistance to biotic stress, environmental adaptability, seed oil content, and seed index of cotton (Table 6).

**Table 6:** Data that support genetic research on ratoon cropping.

**Main aspects**	**Genetic researches**	**Reference**
Heritability	Immotalizing segregated genetic population	De Souza and Da Silv (1987); Jarwar et al. (2018)
	Genetic stability of agronomic traits	Simongulian and Uzakov (1969); Komala et al. (2018g)
Variability	Observing hybridization variation for many years	Kumar et al. (2011); Komala et al. (2018g)
	Observing the variability by γ-mutagenesis	Muhammad et al. (2015)
Diversity	Origin, distribution and evolution of cotton	Wendel et al. (1994); D'Eeckenbrugge and Lacape (2014)
	Resistance to biotic stress	Taware (1990); Seo et al. (2006)
	Environmental adaptability	De Souza and De Holanda (1993)
	Seed oil content and seed index	Gotmare et al. (2004)

### Identification of Perennial Cotton Germplasm Resources

Some wild cotton (perennial cotton) species contain traits superior to those of annual cultivars in terms of growth and development, physio-biochemical characteristics, and stress resistance (Melo, 1952; Stephens, 1965; Simongulian and Uzakov, 1969; Plnheiro et al., 1970; De Souza and De Holanda, 1993). Among them, the perennial allotetraploids in the primary gene pool of *Gossypium* species are most easily applied to the breeding of upland cotton and sea island cotton, and their special agronomic traits are shown in Table 7. For example, *G. hirsutum* ssp. *purpurascens*, a perennial cotton species with year-round flowering and resistance to several biotic and abiotic stresses, is suitable for ratoon cultivation or breeding as a parent (Zhang et al., 2020a).

**Table 7:** Special agronomic traits in perennial allotetraploid cotton species of the primary gene pool.

**Perennial Allotetraploids**	**Special agronomic traits**
Wild races of *G. hirsutum*	Resistances to several biotic and abiotic stresses and other useful traits in *G*. *h*. *mair-galanet, palmerii, morrilli, punctatum, yucatanense, richmonidii, latifolium* (Gao, 2004; Zhang et al., 2020a)
Wild races of *G. barbadense*	Long and high fiber quality, resistance to *Verticillium* wilt in *G*. *b*. *brasiliense, peruriaxum, vitifolium* (Gao, 2004; Zhang et al., 2020a)
*G. tomentosum*	Tolerance to heat stress, source of the nectariless trait, resistance to tarnished plant bug, fleahoppers, boll rot, bollworm, jassids, and thrips, long fiber and high fiber fineness (Shim et al., 2018)
*G. mustelinum*	Long fiber (Wendel et al., 1994)
*G. darwinii*	High fiber quality, resistance to *Verticillium* and *Fusarium* wilt, drought tolerance (Chen et al., 2015)
*G. ekmanianum*	Tolerance to drought and salt stress (Ditta et al., 2018), fiber content is higher than wild coastal cotton (Krapovickas and SEIJO, 2008)
*G. stephensii*	larger than average petal spot in comparison to the other Pacific cottons, dense leaf pubescence (Stephens, 1966), stems pubescent with stellate hairs, lacke extrafloral nectaries at anthesis (Gallagher et al., 2017)

Perennial cotton, an eco-friendly crop for carbon farming solutions (Zhang et al., 2020a), contains significantly different levels of many metabolites compared to annual cotton. As an eco-friendly carbon farming crop, perennial cotton species contain many metabolites different from those of annual cotton species. From a 3-year study carried out in India, of the 20 wild species and 8 accessions of *G. arboretum*, the highest seed oil content was recorded in the wild species *G. lobatum*, followed by *G. harknessii*, which showed that wild perennial species are helpful in improving the seed oil content of cultivated *G. arboreum* (Gotmare et al., 2004). In Brazil, a study found that the starch content in the roots of perennial cotton species was much higher than that in the roots of annual cotton (De Souza and Da Silv, 1987). It is thought that the roots of perennial cotton must reserve sufficient carbohydrates to start a subsequent asexual growth cycle when conditions are suitable or to counter a period of drought stress (Sadras, 1996; Wells, 2002).

### Genetic Research on Ratoon Cotton

Cryotolerance is an essential trait in ratoon cotton breeding (Zhang et al., 2011). To investigate the interspecific heterosis and cytoplasmic effects of cryotolerance-related traits between annual and 2-year-old cotton, four reciprocal crosses of F_1_ hybrids and their parents were used. The results showed that the cold tolerance-related traits of the 2-year-old hybrid F_1_ showed transgressive heterosis, which was better than that of the annual hybrid F_1_; in addition, there was no significant cytoplasmic effect on the cryotolerance-related traits of annual and 2-year-old cotton; however, there was some effect of nuclear–cytoplasm interaction (Zhang et al., 2011). In another study, the mixed genetic model of the major gene plus polygene was used to research the cryotolerance inheritance of cotton in the overwintering period, and the results were consistent with two major additive genes plus the additive dominance polygene genetic model. These results suggested that inbreeding cryotolerant ratoon cotton, single-cross recombination, or single backcrossing would be helpful for transferring major genes associated with overwintering cryotolerance and selection in the F_2_ generation would be efficient (Zhang et al., 2012c,d). With the mining, labeling, cloning, and functional identification of perennial-related genes in wild cotton (Bourgou et al., 2017), more and better possibilities for molecular breeding in cotton will become available.

### Ratooning Perennial Cotton for Breeding

Ratoon cotton can be used for breeding, such as in conserving germplasms, utilizing heterosis, and analyzing combination ability and heritability (Thomson and Luckett, 1988a,b; Komala et al., 2018d,f,g, 2019). Technological systems with great application value could be established to produce hybrid cotton seeds in frost-free areas for sowing cultivation in temperate zones (Zhang et al., 2020a,b). Researcher-based and company-involved strategies for perennially producing hybrid cotton seeds are in the small-area testing phase in southern China, where almost no cotton is currently planted, so cotton plants for producing hybrids can be easily separated spatially, which is conducive to improving the purity of hybrid seeds. Due to the warm environment, ratoon cotton flowers earlier with a more extended boll-opening period than annuals without the need for breeding bees for pollination or hand-pollination due to rich insect sources.

## Future Studies on Ratooning Perennial Cotton for Breeding

### Ratooning Perennial Cotton for Breeding Cytoplasmic Male-Sterile Lines

Male sterility plays an impressive role in heterosis utilization by facilitating hybrid breeding and has contributed greatly to the increased yield of many crops globally (Fan and Zhang, 2018). In cotton, due to limited resources and negative cytoplasmic effects, CMS lines have not been widely used (Zheng et al., 2019; Li et al., 2021). Most of the existing male sterility used in production has the genetic background of wild cotton, such as CMS-D2 and CMS-D8 (Zhang et al., 2019). Some wild cotton lines become sterile after transplanting from the origin (Zhang et al., 2022), showing traits such as non-flowering, non-dehiscence of anthers, and self-incompatibility. Through distant hybridization, doubling, and saturation backcrossing, some CMS mutant lines can be screened out (Zheng et al., 2019). Sometimes CMS mutant plants can be found in the field, but the maintainer line cannot be screened out in a timely manner by test crossing. Thus, the perennialization of mutant plants is very important for breeding CMS lines.

### Ratooning Perennial Cotton for Breeding Photothermosensitive Genic Male-Sterile Lines

Two-line hybrid rice with high yield potential is becoming increasingly popular, and the PTGMS line is one of the essential components for breeding two-line hybrid rice (Barman et al., 2019). In cotton, increasing attention has been given to photothermosensitive male-sterile lines (Zhou et al., 2007; Sekhar and Khadi, 2012). In China, light and temperature conditions in the south and north are very different (Qian and Zhu, 2001). Based on this, a breeding strategy called “planting in temperate regions and breeding in the tropics” was employed for rice (Liang et al., 2020), maize (Eagles and Lothrop, 1994), and sweet potato (Lu et al., 1989). The ideal photothermosensitive male-sterile line is sterile in the breeding area, convenient for hybrid-seed production, and fertile in the planting area to obtain a high yield. Most PTGMS lines are strongly influenced by the environment, and to explore and take advantage of this feature, multiregional planting is necessary. A high temperature always makes the PTGMS sterile lines (Zhang et al., 2007; Mishra, 2013). For cotton, the temperature at the flowering stage needs to be higher than that at other growth stages (Himanshu et al., 2019). To test the sterile/fertile conversion temperature, transplanting the candidate PTGMS line plants in different areas can be conveniently achieved by a perennial cotton ratooning method.

## Conclusion

This study reviews a vital topic within a broader framework for the utilization of perennial germplasm and ratoon cultivation for cotton breeding. Perennial cotton contains a rich diversity of agronomic and stress resistance traits that are important for expanding the economic performance of cotton cultivars. Ratoon cotton can be used to measure the combination ability and heterosis of hybrid combinations, observe the separation of mutagenized populations (Muhammad et al., 2015), and study the performance of a cultivar under different climates and multiple stress conditions for many years (Chamy, 1979). Increased investment is needed for perennial germplasm research, breeding, cultivation, and agroecological research on ratoon cotton. First, breeding efforts should focus on stabilizing the multiyear yield of ratoon cotton and determining the variety adaptability and cropping arrangements suitable for local conditions. Second, proper male-sterile lines for ratoon cultivation in the tropics should be selected or bred. Although ratoon cultivation of cotton has succeeded in some countries such as Brazil, China, and India, due to the lack of evaluation results with commercial production and sales of hybrid cotton seeds from ratooning systems, we can only hypothesize that this method would have good future prospects.

## Author Contributions

XZ, QY, and AK wrote the manuscript. XZ, RZ, JZ, YF, BZ, YJ, and XD revised the manuscript. ZZ, XZ, and AK conceived the idea. All authors read and approved the final manuscript.

## Funding

This research was funded by the National Natural Science Foundation of China (31571600), the Postgraduate Education Reform and Quality Improvement Project of Henan Province (YJS2022ZX22), the Leading Talent Project in Science and Technology Innovation of Central Plain of China (214200510021), and the Program for Innovative Research Team (in Science and Technology) in University of Henan Province, China (21IRTSTHN023).

## Conflict of Interest

The authors declare that the research was conducted in the absence of any commercial or financial relationships that could be construed as a potential conflict of interest.

## Publisher's Note

All claims expressed in this article are solely those of the authors and do not necessarily represent those of their affiliated organizations, or those of the publisher, the editors and the reviewers. Any product that may be evaluated in this article, or claim that may be made by its manufacturer, is not guaranteed or endorsed by the publisher.
